# Jules Gonin and the Nobel Prize: pioneer of retinal detachment surgery who almost received a Nobel Prize in medicine

**DOI:** 10.1186/s40942-017-0106-7

**Published:** 2018-01-15

**Authors:** Fábio Barreto Morais

**Affiliations:** 1 Universidade Federal de São Paulo-Unifesp, São Paulo, Brazil; 2 Hospital de Olhos de Sergipe, Rua Joaquim Goes 88, apt 301, Aracaju, SE 49020130 Brazil

When considering advancements in the treatment of retinal diseases over the last century, it is essential to mention the name of the Swiss ophthalmologist Jules Gonin, a pioneer of retinal detachment surgery. It is likely that retinal specialists will have already heard of the Jules Gonin club, an ophthalmological retina society that aims at promoting exchanges among specialists in vitreo-retino-choroidal diseases and facilitating and supporting research and publications in this specialized field of ophthalmology. This group together with the Retina Society and the Macula Society is one of the three most prestigious retina associations in the world. Throughout this text I will give a brief life history of this brilliant ophthalmologist and how he almost received a Nobel Prize in Medicine.

Jules Gonin was born in Lausanne, the French region of Switzerland, on August 10, 1870. He soon started to exhibit an extraordinary talent for languages and became proficient in many languages. In 1888, he enrolled in the College of Sciences and studied medicine at the University of Lausanne. Dr. Marc Dufour, the director of the Eye Hospital in Lausanne at the time, offered him a post to start his training in 1896. Later, he started several research projects covering diverse topics such as bacterial conjunctivitis, ocular tumors, and hereditary retinopathies. However, it was in 1902 that Dr. Dufour entrusted him with writing a chapter for the French Encyclopedia of Ophthalmology. This chapter dealt with the problem of retinal detachment. Between 1903 and 1918, Gonin persevered with his studies on the pathogenesis and treatment of retinal detachment. In 1903, at the age of 33 years, Gonin was promoted to *Privat*-*Docent*, which implied considerable teaching commitments. In 1908, he was one of the co-founders of the Swiss Ophthalmological Society and he also became its first president. In 1918, Jules Gonin was selected as the director of the Eye Hospital in Lausanne, and 2 years later, in 1920, he was appointed as the Professor of Ophthalmology at the University of Lausanne [1].

Before the turn of the 20th century, eyes with a retinal detachment were considered to be incurable. At this time, the treatment of retinal detachment was still in its infancy and the surgical treatment success rates were < 5%. From 1902 to 1921, Jules Gonin almost singlehandedly changed the landscape of retinal detachment surgery for ever. He recognized that a retinal break was the cause of retinal detachment and not the consequence, as was largely believed at the time, and that treatment must comprise closure of the break by cauterization. He named the procedure as ignipuncture because he cauterized the retina through the sclera with a very hot pointed instrument. Despite rigorously detailed clinical observations and increasing success rates, his discovery was not readily accepted and sometimes openly opposed by a large section of the ophthalmic establishment. It was not until 1929 that he received worldwide acclaim at the International Ophthalmological Congress in Amsterdam for his surgical technique. His legacy lives on in the Eye Hospital in Lausanne that bears his name and in the Gonin Medal awarded by the International Council of Ophthalmology every 4 years for the highest achievement in ophthalmology [2].

The Nobel Prize Committee considered giving their award to Gonin. A questionnaire on Gonin’s work was sent to several ophthalmic authorities worldwide [3]. All replied favorably, with one exception, the prominent Alfred Vogt, one of the three describers of the Vogt–Koyanagi–Harada disease and famous for his gift of observation and his enormous working capacity, but also for his aggressive nature [4]. Vogt quite wrongly and groundlessly cast doubt on Gonin’s priority in discovering this first consistently successful surgery for treating retinal detachment. This unfortunate decision persuaded the Nobel committee to postpone their decision on Gonin’s work for a year. This has become one of the more glaring omissions for the Nobel Prize. It is possibly that the prize would have been conferred to Gonin the year after because the reasons for the opposition against him receiving the prize were unsubstantiated. Therefore, Jules Gonin nearly received the award for his innovations in retinal detachment surgery; however, his premature death prevented him from receiving the award [5].

Jules Gonin’s last few years of life were exceedingly exhausting. He was inundated with difficult cases from around Europe. Often, these were patients with only one eye. Despite increasing weariness in his final years, he decided to publish his book on retinal detachment surgery called as “Le dιcollement de la rιtine.” Jules Gonin unexpectedly died in late May, 1935. In his will, he stipulated that a large part of his assets will go to the blind who had lost their sight late in life [2]. Today, many young surgeons grow up with the latest vitreoretinal technology; however, they are often no longer aware of previous treatment approaches. The view of the past is essential to understand our current approach, identify as of yet unmet needs, and search for their future solutions [6].
